# Decortication in the Surgical Management of Complete Atypical Femoral Fractures: A Strategy to Accelerate Fracture Healing

**DOI:** 10.3390/jcm15020436

**Published:** 2026-01-06

**Authors:** Young-Ho Cho, Changhun Lim, Dongha Kim

**Affiliations:** Department of Orthopedic Surgery, Daegu Fatima Hospital, 99 Ayang-ro, Dong-gu, Daegu 41199, Republic of Korea; chlim56@naver.com (C.L.); tyra629@naver.com (D.K.)

**Keywords:** atypical femoral fracture, bisphosphonate, decortication, bone union, intramedullary nailing

## Abstract

**Background/Objectives:** Surgical management of atypical femoral fractures (AFFs) stabilized with intramedullary (IM) nailing is frequently challenged by delayed union or nonunion due to the severely suppressed bone turnover characteristic of bisphosphonate-related bone pathology, often leading to a hypertrophic nonunion-like state at the fracture site. This consecutive case series aimed to evaluate the effectiveness of intraoperative percutaneous decortication at the hypertrophic cortex in promoting rapid bone healing in complete AFFs. **Methods:** This was a single-center consecutive case series of patients with complete atypical femoral fractures (AFFs) treated with intramedullary nailing and adjunctive percutaneous decortication since February 2021. The standardized surgical protocol—including percutaneous decortication performed through a small anterolateral incision using an osteotome to create bone chips and stimulate the sclerotic cortex—was applied prospectively to all consecutive patients from February 2021. Of the 20 patients who underwent surgery during this period, 14 with sufficient radiographic follow-up were included in the final retrospective analysis. Data collected included patient demographics, duration of bisphosphonate use, fracture location (diaphyseal vs. subtrochanteric), operative details (including iatrogenic fracture), and radiographic bone union time. Bone union was assessed on serial radiographs by two independent observers. **Results:** All 14 patients were female, with a median age of 75 years (IQR 67–79 years). Thirteen patients (92.9%) had prior bisphosphonate exposure for a median of 4.5 years (IQR 3–10 years). Six fractures were subtrochanteric fractures, and six were complicated by iatrogenic fracture during nail insertion. Postoperative teriparatide was administered to six patients. Radiographic bone union was achieved in all 14 patients at a median of 19 weeks (IQR 16–22 weeks; range 16–24 weeks). No major complications (infection, implant failure, nonunion, or neurovascular injury) occurred during follow-up. **Conclusions:** Percutaneous decortication is a simple, safe, and biologically plausible adjunct to intramedullary nailing. In this series of 14 elderly women with long-term bisphosphonate exposure (median 4.5 years), the technique was associated with 100% radiographic union at a median of 19 weeks without major complications, suggesting a promising strategy that warrants validation in larger, controlled trials.

## 1. Introduction

Bisphosphonates (BPs) have been used globally for several decades as a primary therapeutic intervention for osteoporosis, demonstrating efficacy in increasing bone mineral density and significantly reducing the risk of common osteoporotic fragility fractures, as evidenced by major randomized controlled trials [1,2,3,4]. However, the long-term use of BPs has been increasingly associated with rare yet potentially catastrophic complications, primarily atypical femoral fractures (AFFs) [5,6,7].

The fundamental pathogenesis of BP-related AFFs is widely accepted to be linked to the severe suppression of osteoclast-mediated bone turnover [8]. This chronic inhibition disrupts the normal process of bone remodeling, which is crucial for the removal and repair of accumulated microdamage (fatigue damage) that naturally occurs in the bone, particularly in high-stress areas like the lateral tensile cortex of the femur. This prolonged accumulation of microdamage, often preceded by weeks or months of prodromal pain, ultimately progresses to an incomplete stress fracture [9]. The bone’s inadequate attempt at repair often results in a distinct radiographic sign: focal cortical thickening, characterized by significant periosteal and endosteal hypertrophy. This hypertrophy defines a unique and challenging biological state—a biologically inert, hypertrophic nonunion-like state—that is inherently highly resistant to achieving complete bone healing, especially after the fracture progresses to a complete break [10].

Surgical intervention, typically involving intramedullary (IM) nailing, remains the gold standard for stabilizing complete AFFs. However, compared to typical traumatic femoral fractures (e.g., subtrochanteric or shaft fractures), complete AFFs are notorious for exhibiting prolonged healing times and high rates of complications, including delayed union, nonunion (reported incidence ranging significantly from 22% to 56.5% in the literature), and subsequent fixation failure [11,12,13,14,15]. This profound diminishment in healing capacity is directly attributed to the severe, long-term BP-induced reduction in osteoclastic activity, which is an essential prerequisite for the initial steps of fracture healing, including absorption of necrotic material at the fracture site and the initiation of callus formation [8]. Given these challenges, the use of biological adjuncts, such as the anabolic agent teriparatide, has been explored to enhance fracture healing; yet, its definitive efficacy remains a topic of ongoing debate and inconsistent clinical results [16,17].

The decortication technique, originally introduced by Judet, is a well-established principle in orthopedic surgery [18]. It is widely recognized as an effective and powerful method to mechanically and biologically expose and stimulate sclerotic fracture sites, such as those seen in chronic nonunion, thereby biologically enhancing the healing environment by releasing osteogenic cells, growth factors, and promoting vascularization [18,19,20]. Recognizing the unique pathobiology of the AFF fracture site—specifically its hypertrophic yet biologically inactive sclerotic cortex—we hypothesized that direct, local stimulation of this cortex would serve as a powerful biological catalyst, thereby significantly promoting and accelerating bone union.

The primary objective of this case series was to assess the effectiveness of percutaneous decortication—a minimally invasive technique involving the creation of small, viable bone chips along the hypertrophic anterolateral cortex—to determine whether this surgical adjunct can promote reliable and rapid bone union in complete AFFs stabilized with IM nails.

## 2. Materials and Methods

### 2.1. Patients and Ethical Approval

This study involved a retrospective analysis of data from patients treated according to a prospectively applied standardized surgical protocol. The study was reviewed and approved retrospectively by the Institutional Review Board (IRB) of Daegu Fatima Hospital (IRB No. DFH 2025-04-002). A waiver of additional informed consent for the retrospective chart review and database analysis was granted, as all data were anonymized, de-identified, and the study posed minimal risk to patients.

Ethical Consideration: All patients admitted to our hospital signed a general consent form allowing their medical data to be used for research purposes. Data from patients who declined this consent were excluded from the study, ensuring patient autonomy. Furthermore, the surgical techniques used in this study, including percutaneous decortication, are established procedures [18,19,20] and were judged not to pose any potential harm or undue risk beyond that associated with standard IM nailing. All patients provided specific written informed consent for the surgical procedure, during which the operative method and its rationale were fully explained. Therefore, the authors concluded that there were no ethical issues with the conduct of this study or the use of the patient data.

This was a single-center consecutive case series of patients who underwent surgical treatment for complete atypical femoral fractures (AFFs), including both subtrochanteric and diaphyseal locations, resulting from low-energy trauma. The standardized surgical protocol (intramedullary nailing with adjunctive percutaneous decortication) was applied prospectively to all consecutive patients starting in February 2021, whereas patient identification, data collection, and outcome analysis were performed retrospectively. Low-energy trauma was defined as a fall from standing height or less, or simple tripping. AFFs were diagnosed according to the 2013 revised ASBMR Task Force criteria [21].

Exclusion criteria for the study included fractures treated with plates and screws, incomplete fractures, pathological fractures due to tumor metastasis, and any periprosthetic or peri-implant fractures.

**Figure 1:**
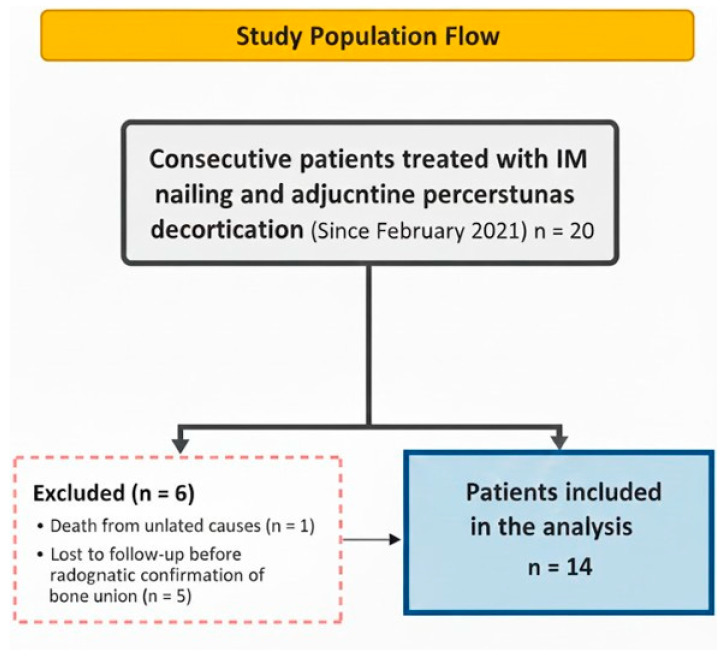
Flow diagram of patient inclusion and exclusion in the study.

Femoral recon nail (FRN; Synthes, Solothurn, Switzerland) was used for fixation in the initial cohort of 20 patients. From February 2021 onward, the standardized surgical protocol (intramedullary nailing with adjunctive percutaneous decortication) was applied prospectively to all consecutive patients with complete atypical femoral fractures. Of these 20 patients, six were excluded from the final analysis: one patient died from causes unrelated to the fracture or surgery, and five were lost to follow-up before radiographic confirmation of bone union. Thus, 14 patients with sufficient radiographic follow-up were included in the analysis (Figure 1).

Follow-up clinical and radiographic assessments were conducted at stringent four-week intervals until radiological evidence of bone union was observed. Bone union was defined according to criteria of Corrales et al.: the clinical absence of pain at the fracture site on palpation and full weight-bearing, combined with radiological evidence showing bridging callus formation in at least three cortices on two different radiographic views (anteroposterior and lateral) [22].

### 2.2. Surgical Procedures

All operations were performed by a single senior orthopedic trauma surgeon (Y.H. Cho) with more than 15 years of experience in complex hip and femoral fracture surgery. Surgery was conducted under general anesthesia with the patient positioned supine on a radiolucent fracture table to allow unrestricted biplanar fluoroscopic imaging.

Standard closed reduction was attempted first in all cases. The intramedullary canal was over-reamed by 2.5 mm relative to the selected nail diameter to achieve an adequate press-fit and to generate reaming debris that could act as local autograft. Regardless of whether the fracture was subtrochanteric or diaphyseal, a long cephalomedullary nail was used and two cephalomedullary screws were routinely inserted into the femoral head and neck. Distal locking was performed using an angular-stable locking system (ASLS) in the majority of cases to enhance rotational and axial stability.

Standardized percutaneous decortication protocol (performed in all 14 patients).

Immediately after proximal locking, traction was released to bring the fracture ends into close apposition. Percutaneous decortication was then performed as the final step before distal locking and wound closure. A 1.5–2.0 cm longitudinal skin incision was made on the anterolateral aspect of the thigh directly overlying the fracture site under fluoroscopic guidance. Minimal subcutaneous dissection was performed to reach the iliotibial band, which was split in line with its fibers.

A 10-mm-wide curved osteotome was introduced percutaneously. Under C-arm control (strict lateral and anteroposterior views), the tip of the osteotome was positioned exactly at the junction between the thickened, sclerotic cortices of the proximal and distal fragments. The osteotome was intentionally angled 30–45° to the long axis of the femur (i.e., nearly perpendicular to the cortical surface) and gently malleted 5–8 mm deep until definite penetration of the thick sclerotic cortex was felt and confirmed fluoroscopically by sudden loss of resistance

**Figure 2:**
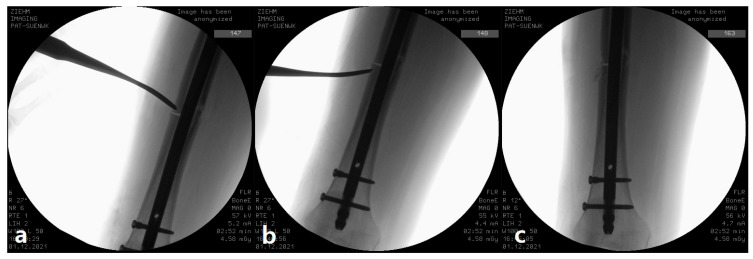
Intraoperative photographs demonstrating decortication after intramedullary nail fixation. (**a**) Proximal decortication is performed using a curved osteotome, (**b**) Distal decortication of the lateral cortex, (**c**) Fracture site after completion of decortication.

Decortication was systematically performed over a total length of 3 cm centered on the fracture line (1.5 cm proximal and 1.5 cm distal to the fracture). In each 1.5 cm segment, 4–6 separate bone chips were created, yielding a total of 8–12 chips per patient. Each chip size was approximately 3–5 mm in length (Figure 2). The goal was to transform a biologically inert sclerotic “beak” into multiple small, vascularized surfaces. This meticulous percutaneous approach was purposefully chosen to minimize the risks associated with traditional open procedures, specifically surgical site infection and excessive stripping of the periosteum, thereby preserving the critical surrounding soft-tissue and vascular envelope.

### 2.3. Postoperative Medical Treatment and Rehabilitation

Postoperatively, bisphosphonate therapy was universally discontinued in all patients, and a standardized regimen of calcium and vitamin D supplementation was immediately initiated. Teriparatide (recombinant human PTH 1–34, 20 µg daily subcutaneous injection) was routinely recommended to all patients as an off-label anabolic adjunct to potentially enhance healing of atypical femoral fractures. Patients were verbally informed that the drug is approved only for osteoporosis treatment, that its use for fracture healing is off-label and not supported by high-level evidence, and that the entire cost would be borne by the patient because it is not reimbursed by national health insurance for this indication. After this explanation, teriparatide was prescribed for a fixed duration of 3 months only to those patients who fully understood the off-label nature and explicitly agreed to self-fund the treatment.

Prophylaxis against deep vein thrombosis involved the use of intermittent pneumatic compression devices, but no chemical prophylaxis was routinely administered.

Rehabilitation protocols commenced on the third day post-surgery, or as soon as acute pain management allowed. The focus was on strengthening exercises for the quadriceps and maintaining a full range-of-motion (ROM) for the hip and knee joints. Patients were actively encouraged to participate in out-of-bed activities using a wheelchair for mobility. Crucially, weight-bearing was strictly restricted and monitored until bone union was definitively confirmed radiographically according to the defined criteria.

### 2.4. Statistical Analysis

Statistical analyses were performed using SPSS version 29.0 (IBM Corp., Armonk, NY, USA) or R statistical software version 4.3.2. Given the small sample size (total N = 14) and the non-normal distribution of bone union time (verified by Shapiro–Wilk test and visual inspection of histograms), all continuous data are presented as medians with interquartile ranges (IQR). Categorical variables are reported as frequencies and percentages. Between-group differences in bone union time were evaluated using the exact Mann–Whitney U test. Effect sizes are reported as Hodges–Lehmann median differences with corresponding 95% confidence intervals (CI). All statistical tests were two-sided, and *p*-values < 0.05 were considered statistically significant.

## 3. Results

The baseline characteristics of the 14 patients are summarized in Table 1.

All patients were female, with a median age of 75 years (IQR 67–79 years) and a median body mass index of 24.1 kg/m^2^ (IQR 23.3–25.6 kg/m^2^). Thirteen patients (92.9%) had been receiving bisphosphonates for a median of 4.5 years (IQR 3–10 years) prior to the fracture. Six fractures (42.9%) were subtrochanteric and eight (57.1%) were diaphyseal; six (42.9%) were considered iatrogenic. Postoperative teriparatide was administered to six patients (42.9%). The median Charlson comorbidity index was 1 (IQR 0–1), with most patients having a score of 0 (n = 4) or 1 (n = 7). Bone mineral density T-scores were a median of −2.25 (IQR −2.3 to −1.9) at the lumbar spine and −2.75 (IQR −3.3 to −1.9) at the total hip. The median postoperative follow-up duration was 9 months (IQR 7–13 months).

Bone union was achieved in all 14 patients at a median of 19 weeks (IQR 16–22 weeks).

Table 2 summarizes bone union time according to fracture location, presence of iatrogenic fracture, and teriparatide use.

Due to the small sample size and non-normal distribution of union times, results are presented as medians with interquartile ranges. Differences between groups were estimated using the Hodges–Lehmann median difference with 95% confidence intervals, and statistical significance was assessed with the exact Mann–Whitney U test.

Subtrochanteric fractures showed a median union time 4 weeks longer than diaphyseal fractures (median 20 vs. 18 weeks; median difference −4 weeks, 95% CI −8 to 1 weeks; *p* = 0.062).

Patients with iatrogenic fractures required a median of 20 weeks compared with 17 weeks in those with non-iatrogenic fractures (median difference 4 weeks, 95% CI −1 to 8 weeks; *p* = 0.181).

Teriparatide users had a median union time of 20 weeks versus 19 weeks in non-users (median difference 1 week, 95% CI −5 to 7 weeks; *p* = 0.573).

All 95% confidence intervals were wide and included zero, and none of the examined factors reached statistical significance.

A detailed subgroup analysis using the non-parametric Mann–Whitney U test revealed no statistically significant difference in the duration of bone healing based on several critical variables. Specifically, fracture location (Diaphysis vs. Subtrochanteric) showed no significant difference (*p* = 0.062). The occurrence of an iatrogenic fracture during IM nail insertion—which occurred in six cases (42.8%)—did not significantly impact the overall healing time (*p* = 0.181). Furthermore, the administration of teriparatide postoperatively (N = 6, 42.8%) was not associated with a statistically significant difference in median bone union time compared to those who did not receive it (*p* = 0.573).

Iatrogenic fractures occurred during nail insertion in six patients; however, these were stabilized by the IM nail and healed uneventfully. No other major surgical complications were observed, specifically excluding infection, neurovascular injury, hardware failure, or secondary peri-implant fractures during the follow-up period. Among the patients who received teriparatide, no adverse events directly attributable to the medication were documented during the course of treatment. All patients demonstrated a standard, uneventful recovery without any adverse postoperative outcomes attributable to the decortication procedure.

## 4. Discussion

The introduction of BPs has revolutionized osteoporosis management, successfully reducing fragility fractures [1,2,3,4]. However, the critical problem remains the small but serious risk of AFFs associated with long-term use [5,6,7]. The key pathological challenge in AFFs is not the severity of the initial trauma but the compromised bone biology—the accumulation of microdamage and the resultant hypertrophic nonunion-like state of the sclerotic anterolateral cortex, which fundamentally resists normal fracture healing due to profoundly reduced bone turnover [10]. Reduced healing capacity directly leads to unfavorable clinical outcomes post-surgery. Literature consistently reports concerning rates of nonunion or delayed union following IM nailing for complete AFFs, with figures ranging widely but frequently exceeding 20% [11,12,13,14,15]. The reported time to bone union after treating complete AFFs with IM nailing varies widely, ranging from 6 to 31 months [11,12,13,14]. Optimizing surgical outcomes, including achieving adequate reduction (particularly minimizing the gap in the anterolateral cortex) and avoiding malalignment, is emphasized as critical [15,23]. Even before the adoption of decortication, instances of nonunion and delayed union were observed in patients treated at our center, despite strict adherence to the surgical principles outlined above (unpublished data). The median time to union in our cohort was 19 weeks (IQR 16–22 weeks; approximately 4.4 months), with 100% union achieved within 6 months. This substantial reduction in healing time compared to previously reported periods—achieving union in less than half the time—provides strong evidence that the percutaneous decortication technique imparts a powerful and effective biological stimulus.

The rationale for employing decortication (the Judet technique) is deeply rooted in stimulating bone’s intrinsic regenerative capacity [18,19,20]. The procedure, as applied here, serves two critical biological functions [20]

Osteoinduction and Osteogenesis: The most significant effect is the conversion of the pathologically inert, sclerotic cortex into an actively healing surface. The intentional creation of bone chips and the penetration of the cortex effectively expose the bone marrow and the highly vascularized periosteal layer. This action immediately releases a concentrated local milieu rich in osteogenic stem cells, growth factors (e.g., BMPs, TGF-ß), and fresh vascularity. By circumventing the BP-suppressed remodeling cycle, this massive influx of pro-healing elements is thought to overcome the local biological inertia and directly initiate a more vigorous callus formation.

**Figure 3:**
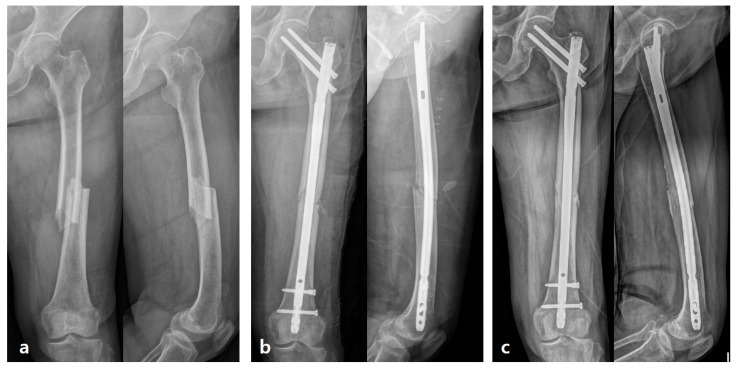
Serial anteroposterior and lateral radiographs of a 77-year-old woman. (**a**) Preoperative radiographs show a diaphyseal complete atypical femoral fracture, (**b**) Immediate postoperative radiographs after fixation with an IM nail and decortication at the fracture site, (**c**) Radiographs at 4 months postoperatively demonstrate complete cortical bridging and fracture union.

**Figure 4:**
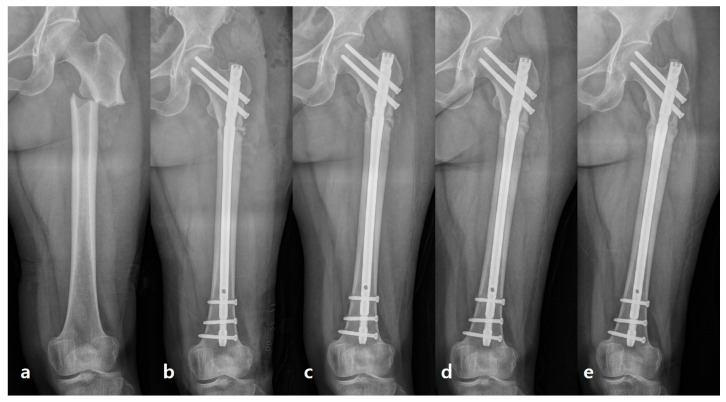
Serial anteroposterior radiographs of a 60-year-old woman with a subtrochanteric complete atypical femoral fracture fixed with femoral recon nail. (**a**) preoperative, (**b**) immediate postoperative, (**c**) 6 weeks follow-up, (**d**) 14 weeks follow-up, (**e**) 22 weeks follow-up X-ray shows progressive callus formation and consolidation of decorticated bone chips.

Mechanical Strain Modification: While fixation with an IM nail provides primary stability, transverse fracture line of the sclerotic AFF cortex can be subject to high localized mechanical strain. By creating small controlled fractures (the bone chips), the procedure essentially converts a simple, high-strain fracture interface into a multi-fragmented low strain surface. This acts to relieve highly concentrated mechanical strain at the problematic main fracture line (anterolateral cortex), facilitating easier callus formation and bridging (Figure 3 and Figure 4).

The potential role of postoperative teriparatide in atypical femoral fractures remains controversial. Although its strong anabolic effect has led to widespread off-label use and several observational studies have suggested faster healing, high-quality randomized evidence is still lacking, and a recent meta-analysis concluded that its efficacy in accelerating AFF healing remains inconclusive [17,24].

In the present study, teriparatide (20 μg daily subcutaneous injection for 3 months) was routinely recommended to every patient as an optional biological adjunct. Because the drug is not reimbursed by national health insurance for this indication in our country, the final decision was made after thorough verbal explanation of the potential benefits, absence of strong evidence, and full out-of-pocket cost. Treatment was administered only to patients who verbally agreed and were willing to bear the entire expense. Consequently, only 6 of the 14 patients received teriparatide, resulting in a non-randomized subgroup driven exclusively by financial considerations and unrelated to clinical or fracture characteristics.

Given the very small numbers and non-random allocation, the subgroup comparison is exploratory only and severely underpowered. We observed no significant difference in median bone union time between patients who did (20 weeks, IQR 16–24) and did not (19 weeks, IQR 16–20) receive teriparatide (*p* = 0.573; Hodges–Lehmann median difference 1 week, 95% CI −5 to 7 weeks). Importantly, all 14 patients achieved radiographic union within 6 months regardless of teriparatide use.

These results suggest that, when percutaneous decortication provides immediate and targeted local biological stimulation at the sclerotic fracture site, systemic teriparatide may not offer substantial additional acceleration of radiographic healing in complete AFFs. However, due to the limitations described above, no definitive conclusions can be drawn about the efficacy of teriparatide in this setting. Larger, randomized trials are needed to determine whether teriparatide provides meaningful additive benefit beyond optimized surgical techniques that directly address the local biological impairment of AFFs.

Furthermore, unlike the open bone end procedure described by Canbek et al. [25], which necessitated a larger surgical exposure (and still reported a case of nonunion), our procedure is percutaneous. This is a major advantage. By targeting the hypertrophic cortex under C-arm guidance without direct exposure of the main fracture site, we significantly minimize surgical morbidity. This preservation of the overlying soft tissue and vascular envelope is paramount in the compromised biological environment of BP-treated bone, substantially reducing the risk of surgical site infection and maximizing local vascular contribution to healing, which are major concerns in any open surgery for nonunion.

Our subgroup analysis demonstrated that neither fracture location (subtrochanteric vs. diaphyseal) nor the presence of an iatrogenic fracture during surgery significantly impacted the achieved union time. The high rate of iatrogenic fractures (42.9%) reflects the inherently fragile and brittle nature of the BP-treated bone, which is poorly suited to withstand the hoop stresses of reaming and nail insertion. Furthermore, the geometry of the femur, particularly the presence of significant anterior or lateral femoral bowing, is a known risk factor for iatrogenic fracture during the placement of an IM nail in the stiffened AFFs cortex. The mismatch between the nail and a highly bowed canal can concentrate stress, leading to a fracture during the insertion of IM nail. However, the fact that these complications did not prolong the healing time confirms that the IM nail provided sufficient mechanical stability, and the decortication provided adequate biological input to overcome these complicating factors. This underscores the effectiveness of the integrated fixation and biological stimulation approach.

This study has several important limitations, and the results should be interpreted with caution.

First, although the percutaneous decortication technique was applied prospectively according to a standardized institutional protocol in all consecutive patients, data collection and outcome analysis were performed retrospectively once an adequate number of cases had been accumulated. The study therefore retains the inherent limitations of retrospective assessment.

Second, the sample size is small (N = 14), and loss to follow-up reached 25%. Patients who experienced complications or delayed healing may have sought care elsewhere, which could make the observed union rate appear artificially higher.

Third, because the primary focus of this study was radiographic union time and surgical complications, functional outcomes and patient-reported measures (pain, mobility, quality-of-life, etc.) were not analyzed. Their absence is an important limitation in this elderly female cohort.

Fourth, although the study utilized a prospectively collected database, there was no contemporary control group treated with standard IM nailing alone. After observing accelerated healing in the initial cases, we applied percutaneous decortication universally to subsequent patients. Nevertheless, it is worth noting that prior to the routine use of decortication at our institution, we encountered several cases of delayed union and nonunion despite strict adherence to standard surgical principles (unpublished institutional data). This historical experience suggests that the 100% union rate observed in the current series may not be explained by bias alone but could also reflect a genuine biological effect of decortication in the setting of atypical femoral fracture pathology.

Given the incomplete follow-up, small sample size, lack of a control group, and absence of functional outcomes, the reported 100% radiographic union rate must be interpreted cautiously. Larger, fully prospective, and preferably randomized controlled trials—comparing percutaneous decortication plus intramedullary nailing against standard nailing without cortical intervention and incorporating both radiographic and patient-centered outcomes—are needed to validate these findings.

## 5. Conclusions

The percutaneous decortication technique applied to the sclerotic anterolateral hypertrophic cortical bone may serve as a feasible, minimally invasive, and biologically supportive adjunct to standard IM nail fixation. By directly addressing the biologically inert fracture environment, this technique has the potential to promote more favorable conditions for healing. Although the observed outcomes are encouraging, the small sample size and absence of a control group limit the generalizability of these preliminary findings. Further studies with larger cohorts and comparative designs are warranted to validate the clinical usefulness and reproducibility of this approach in the management of complete AFFs.

## Figures and Tables

**Table 1:** Basic demographics of all subjects.

Characteristic	Value
Age, years	75 (67–79)
Female sex	14 (100%)
Body mass index, kg/m^2^	24.1 (23.3–25.6)
Follow-up duration, months	9 (7–13)
Duration of bisphosphonate use, years	4.5 (3–10)
Bisphosphonate use	
Yes	13 (92.9%)
No	1 (7.1%)
Fracture location	
Subtrochanteric	6 (42.9%)
Diaphyseal	8 (57.1%)
Iatrogenic fracture	
Yes	6 (42.9%)
No	8 (57.1%)
Teriparatide use after surgery	
Yes	6 (42.9%)
No	8 (57.1%)
Charlson comorbidity index	
0	4 (28.6%)
1	7 (50.0%)
3	2 (14.3%)
5	1 (7.1%)
Bone mineral density, T-score	
Lumbar spine	−2.25 (−2.3 to −1.9)
Total hip	−2.75 (−3.3 to −1.9)

**Table 2:** Comparison of bone union time according to variables.

Variable	Subgroup	N	Bone Union Time, Median (IQR), Weeks	Hodges–Lehmann Median Difference (95% CI), Weeks	*p*-Value
All patients		14	19 (16–22)	–	–
Fracture location	Diaphysis	8	18 (16–22)	−4 (−8 to 1)	0.062
	Subtrochanteric	6	20 (18–22)	–	
Iatrogenic fracture	Yes	6	20 (18–24)	4 (−1 to 8)	0.181
	No	8	17 (16–20)	–	
Teriparatide use	Yes	6	20 (16–24)	1 (−5 to 7)	0.573
	No	8	19 (16–20)	–	

N: number of patients. *p*-value from Mann–Whitney U test. IQR: interquartile range; CI: confidence interval.

## Data Availability

The datasets generated and/or analyzed during the current study are not publicly available because of restricted access to our hospital database but are available from the corresponding author upon reasonable request.

## Abbreviations

The following abbreviations are used in this manuscript:

AFFs	Atypical femoral fractures
BPs	Bisphosphonates
IM	Intramedullary
ASBMR	American Society for Bone and Mineral Research
FRN	Femoral recon nail
ASLS	Angular stable locking system
ROM	Range of motion
